# Computational modeling of airway instability and collapse in tracheomalacia

**DOI:** 10.1186/s12931-017-0540-y

**Published:** 2017-04-19

**Authors:** Scott J. Hollister, Maximilian P. Hollister, Sebastian K. Hollister

**Affiliations:** 1 Wallace A. Coulter Department of Biomedical Engineering, Georgia Institute of Technology, Rm 2102 UA Whitaker Biomedical Engineering Bldg, 303 Ferst Drive, Atlanta, GA 30332 USA; 2 Department of Biomedical Engineering, The University of Michigan, Ann Arbor, MI USA

## Abstract

**Background:**

Tracheomalacia (TM) is a condition of excessive tracheal collapse during exhalation. Both acquired and congenital forms of TM are believed to result from morphological changes in cartilaginous, fibrous and/or smooth muscle tissues reducing airway mechanical properties to a degree that precipitates collapse. However, neither the specific amount of mechanical property reduction nor the malacic segment lengths leading to life threatening airway collapse in TM are known. Furthermore, the specific mechanism of collapse is still debated.

**Methods:**

Computational nonlinear finite element models were developed to determine the effect of malacic segment length, tracheal diameter, and reduction in tissue nonlinear elastic properties on the risk for and mechanism of airway collapse. Cartilage, fibrous tissue, and smooth muscle nonlinear elastic properties were fit to experimental data from preterm lambs from the literature. These elastic properties were systematically reduced in the model to simulate TM.

**Results:**

An intriguing finding was that sudden mechanical instability leading to complete airway collapse occurred in airways when even a 1 cm segment of cartilage and fibrous tissue properties had a critical reduction in material properties. In general, increased tracheal diameter, increased malacic segment length coupled with decreased nonlinear anterior cartilage/fibrous tissue nonlinear mechanical properties increased the risk of sudden airway collapse from snap through instability.

**Conclusion:**

Modeling results support snap through instability as the mechanism for life threatening tracheomalacia specifically when cartilage ring nonlinear properties are reduced to a range between fibrous tissue nonlinear elastic properties (for larger diameter airways > 10 mm) to mucosa properties (for smaller diameter airways < 6 mm). Although reducing posterior tracheal smooth muscle properties to mucosa properties decreased exhalation area, no sudden instability leading to collapse was seen in these models.

## Background

Tracheomalacia (TM) has been noted as the most prevalent of tracheal pathologies, affecting 1 in 2100 live births [1, 2]. TM may be congenital or due to abnormal mechanical compression of the trachea from surrounding tissues [1, 3]. The mechanism of collapse is attributed to reduced tracheal cartilage mechanical stiffness, potentially in conjunction with reduced posterior smooth muscle mechanical stiffness [1, 3–5].

Initial pediatric tracheal mechanical properties that support healthy respiration and the degree of tracheal mechanical stiffness reduction initiating tracheal collapse and respiratory difficulties are largely unknown. Animal studies suggest the pediatric trachea is initially very compliant due a hypercellular matrix [5–8]. Animal studies also show the growing airway stiffens due to developmental changes in airway geometry increasing the ratio of cartilage to soft tissue in conjunction with increases in cartilage glycosaminoglycans that stiffen the cartilage rings [7, 8]. These events suggest an increasingly stiffer trachea is necessary to avoid excessive airway deformation and collapse under expiration as the airway grows.

Questions remain, however, as to how malacic segment length, reduction in airway mechanical properties, and airway diameter contribute to the risk of trachea collapse in TM. Such effects, especially mechanical properties, are difficult to quantitatively assess in patients. Therefore, TM has been studied in animal models by resecting cartilage rings [9–12]. Although these models are extremely valuable for testing TM treatments, they provide limited insight into either the mechanism of airway collapse or how much tissue stiffness must change to induce collapse since basically the cartilage ring stiffness becomes zero if the cartilage ring segment is completely resected.

Determining TM severity dependence on malacia segment length, airway diameter and airway nonlinear mechanical properties would advance diagnosis and treatment of TM. Benjamin et al. [13] classified TM as mild, moderate, and severe, with severe being associated with cyanosis, apneic spells, respiratory distress and stridor [4, 13]. However, difficulty quantifying exhalation pressures or airway mechanical properties in patients or animal models hinders interpretation of these diagnostic criteria.

Given the difficulty in studying the physical mechanism of airway collapse and TM severity in both patients and animal models, computational modeling is a complimentary approach that can provide insight into both TM severity and collapse mechanism. Computational models allow systematic variation in airway diameter, malacic segment length and airway tissue nonlinear mechanical properties to determine how each of these variables affect TM severity and collapse mechanisms. A number of investigators have utilized computational finite element models to study the effects of liquid ventilation, endoprostheses and stenosis on tracheal mechanics [14–21]. The FDA has also recognized the value of computational finite element modeling in obtaining additional information on medical devices that cannot be obtained by bench studies, animal studies, or even human clinical trials ([22], “https://www.fda.gov/downloads/MedicalDevices/DeviceRegulationandGuidance/GuidanceDocuments/UCM381813.pdf”). The current study used computational modeling to examine how variations in tracheal nonlinear elastic mechanical properties, malacic segment length, and tracheal diameter affected both the mechanism and severity of tracheal collapse under exhalation pressure.

## Methods

The finite element method (FEM) was used to analyze tracheal mechanics under exhalation pressures. FEM is a numerical method used to computationally solve the governing stress equilibrium equations for 3D structures. Especially for biological tissues, which exhibit complex nonlinear stress-strain behavior, the finite element method is the most widely used computational method to determine structural displacement, strains and stresses under applied forces.

Nonlinear finite element models of idealized trachea were constructed with a length of 50 millimeters (mm) and semi-circular diameters of 6, 8 and 10 mm to simulate typical tracheal geometry in newborns and children under 1 year of age. The anterior portion of trachea was represented as semi-circular periodic structure with alternating rings of cartilage 2 mm wide separated by soft fibrous tissue 1 mm wide, both of which were 0.8 mm thick, representing an upper bound of pediatric wall thickness compared to known adult tracheal wall thickness [23]. The trachealis muscle was represented as a flat structure attached to the bottom of the cartilage/soft tissue, also with a thickness of 0.8 mm. A layer of mucosal tissue (0.2 mm thick) was assumed to line the semi-circular lumen. Exhalation was modeled as a negative pressure normal to the airway wall ranging from 0 to 40 cm H_2_0. The airway was assumed to undergo large deformation (strains typically greater than 5%) and contact was allowed between the lumen walls on collapse.

A difficulty in any tissue computational model is determining appropriate tissue mechanical properties, also known as constitutive models. Most biologic soft tissues are modeled as either nonlinear elastic or nonlinear viscoelastic. No nonlinear viscoelastic data exists to our knowledge for young humans (<15 years of age) or even young animals. Indeed, very limited nonlinear viscoelastic data exists for human airway, with data published for ages 20 years and older [24]. Most studies model tracheal tissues as nonlinear elastic (hyperelastic), although again the majority of data is from older humans (>20 years old) [14–21, 25]. No nonlinear elastic data to our knowledge exists for children, and limited nonlinear elasticity data exists for young animals [14, 16]. Given the lack of viscoelastic data for young members of any species, the fact that collapse occurs over a short period of time, and the fact that Sakshekan et al. [24] reported that the strain rate dependency of airway tissues changed much less than elastic stiffness (albeit for adult specimens), we chose to model all airway tissues as nonlinear elastic.

Unlike linear elastic behavior that is uniquely characterized by Hooke’s law, there is no unique model for nonlinear elasticity. Nonlinear elastic models are constructed using strain energy functions. We chose to use a two term isotropic Ogden model to characterize tracheal tissue nonlinear elasticity. The Ogden strain energy function is given by:

1 $$ W=\frac{a}{b}\left({\lambda}_1^b+{\lambda}_2^b+{\lambda}_3^b-3\right) $$

Where a and b are material coefficients determined by fitting the model to experimental data and λ_1_, λ_2_ and λ_3_ are the ratios between the deformed length of material to the original length in the 1 (x), 2 (y) and 3(z) directions, respectively. These ratios are denoted as stretch ratios. For tracheal cartilage, fibrous tissue and smooth muscle, we fit nonlinear stress-strain data published by Bagnoli et al [14] for lamb pre-term trachea to the Ogden strain energy function from Eq. 1 using the MATLAB optimization routine fminunc. This paper, however, did not provide data for mucosa tissue. We used Ogden constants for engineered mucosa tissues we previously published as constants for engineered oral mucosa tissue [26].

Tracheomalacia was simulated in the finite element model by sequentially reducing cartilage properties to be the same as the soft fibrous tissue properties interposed between the cartilage rings (denoted as “soft” models) and even further reducing both cartilage and fibrous tissue properties to mucosa tissue properties (denoted as “mucosa” models). The effect of anterior malacic segment length was simulated by sequentially reducing cartilage ring Ogden coefficients to either soft or mucosa Ogden coefficients for 1 ring, 3 ring, 5 ring, or 7 ring segments equivalent to 2, 8, 14, and 20 mm malacic segment lengths. Posterior malacia was simulated by reducing nonlinear trachealis muscle properties to nonlinear properties in between those of smooth muscle and mucosa and to nonlinear properties equivalent to those of mucosa for malacic lengths of 2, 8, 14, 20, 26, and 32 mm. These malacic lengths are within those noted by Morrison et al [27], who used splints ranging in length from 12 to 22 mm to cover malacic segments in three pediatric patients.

All finite element simulations were run using the software FEBio (febio.org) which was written to solve tissue mechanics problems using the finite element method [28]. This code has been validated against both analytical solutions for nonlinear materials and commercial finite element codes [28]. The pre-processor PreView and post-processor PostView were used to generate the initial idealize tracheal models and analyze the resulting deformations. Tracheal models of 6, 8 and 10 mm semi-circular diameter and 50 mm length were constructed with 2 mm wide cartilage rings separated by 1 mm wide fibrous tissue segments.

All simulation displacement and strain data was output at 4 cm H_2_O pressure intervals. Mid-sectional trachea images were captured using PostView and exported in TIFF format. TIFF images were imported to ImageJ, and the internal lumen area at each deformation step was determined using the polygonal outlining tool. All subsequent lumen areas were calibrated to the initial intact tracheal lumen and area ratios were calculated in Excel.

## Results

**Fig. 1:**
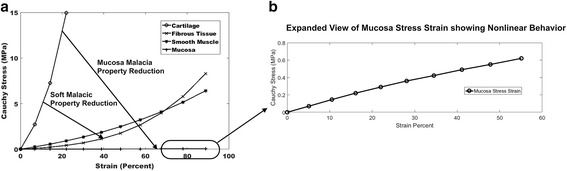
**a** Nonlinear Cauchy stress versus strain curves based on two term Ogden model fits for tracheal cartilage, fibrous tissue, smooth muscle and mucosa tissue. Cauchy stress is defined as the force in the deformed configuration (i.e. after tracheal deformation) divided by the area in the deformed configuration. Drawn arrows represent reduction in nonlinear elastic properties for “soft” malacic models by reducing cartilage ring properties to those of fibrous tissue and “malacic” models by reducing both cartilage and fibrous tissue properties to those of mucosa tissues. Ogden material coefficients for the fit data were as follows: Cartilage a = 0.109, b = 16.63; Fibrous Tissue a = 0.01, b = 11.05; Smooth Muscle a = 0.08; b = 6.29; Musoca a = 0.1 b = 3.4. **b** Expanded view of mucosa stress-strain curve demonstrating nonlinear Neo-Hookean behavior. Mucosa appears flat in Fig. 1a (*left*) due to very low maximum stress (<1 MPa) in mucosa even at 50% strain

The two term Ogden model fit tracheal cartilage, tracheal fibrous tissue and trachealis muscle stress strain values for neonatal lambs [14] well with R^2^ values ranging from 0.968 to 0.995. The average stress strain curves of the Ogden model fits demonstrate the nonlinear elastic behavior of tracheal cartilage, fibrous tissue, smooth muscle and mucosa tissue (Fig. 1).

**Fig. 2:**
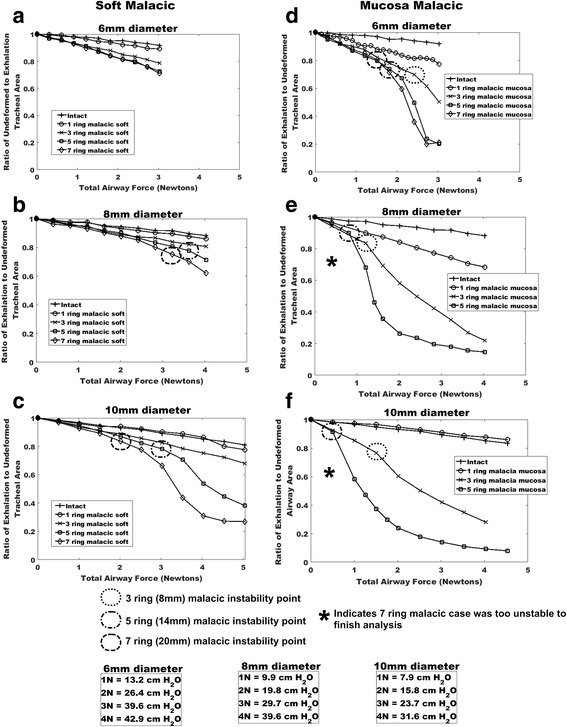
Plots of exhalation to undeformed lumen area ratio for tracheal models of 6, 8 and 10 mm diameter with varying degrees of malacic length (0 to 20 mm) and severity (cartilage/fibrous tissue properties reduced to fibrous tissue - “soft” or mucosa tissue properties -“mucosa”. **a** 6 mm diameter soft malacic. **b** 8 mm diameter soft malacic. **c** 10 mm diameter soft malacic. **d** 6 mm diameter mucosa malacic. **e** 8 mm diameter mucosa malacic. **f** 10 mm diameter soft mucosa malacic. Note that in many cases there is a nearly linear decrease in tracheal area with increasing pressure/airway force. However, in some severe malacic cases (indicated by *dotted or dashed circles*) there is a sharp decrease in tracheal area for little increase in airway pressure/force indicating a “snap through” instability. Lower boxes show conversion between airway force in Newtons and applied exhalation pressure, which varies due to different areas of the 6, 8 and 10 mm diameter models. Finally, cases denoted by * add such severe nonlinear instabilities that the numerical model would not converge to a solution

Plots of tracheal area reduction versus total airway force for 6, 8, and 10 mm diameter airways demonstrate that increasing malacic segment length leads to greater reduction in tracheal area upon exhalation for both the soft malacic models (Fig. 2a-c) and the mucosa malacic models (Fig. 2d-f). Furthermore, decreasing anterior tracheal wall mechanical properties from fibrous tissue to mucosa properties leads to greater reduction in tracheal area during exhalation (compare Fig. 2a to d, b to e and c to f).

**Fig. 3:**
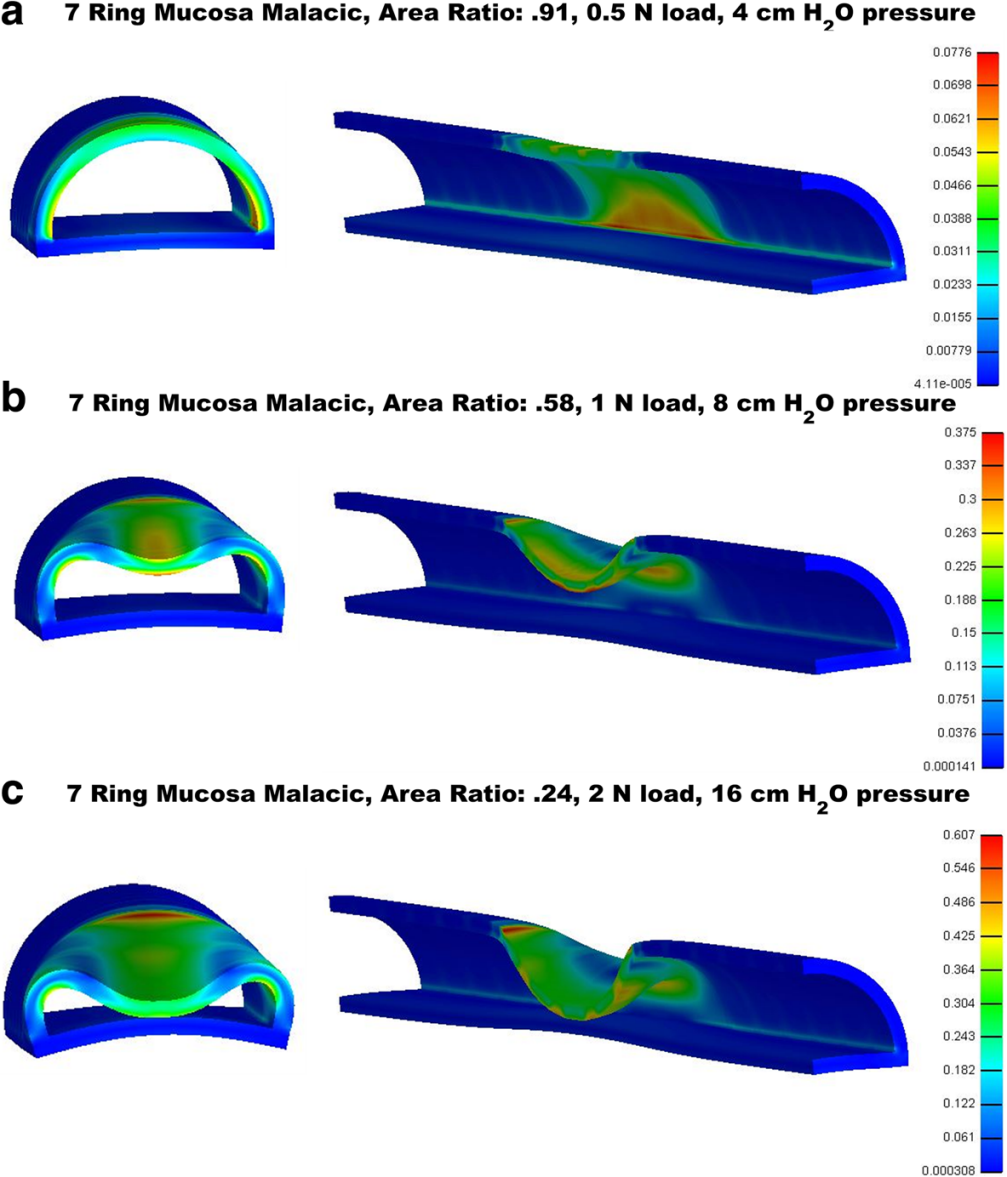
Sequence of instability and collapse in 10 mm diameter “mucosa” model involving large anterior wall displacement under small force increase. Scale bars show decimal value of tracheal wall strain (multiplied by 100 will give percent) **a** immediately prior to instability for 0.5 N force. **b** mid-collapse at 1 N force. **c** near complete collapse at 2 N force constituting severe tracheomalacia with wall strains reaching over 45% and opposing walls contacting during collapse

**Fig. 4:**
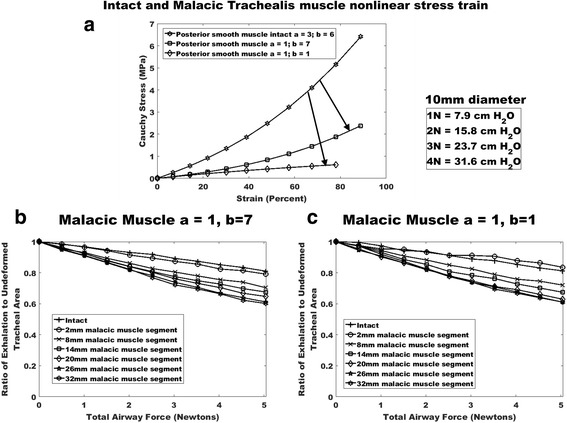
Reduction in trachealis muscle nonlinear elastic properties to simulate tracheal posterior wall weakening. **a** Intact muscle nonlinear elastic Ogden fit with arrows indicating initial reduction in properties to first a = 1, b = 7 from eq. 1 and subsequently to a = 1, b = 1 from eq. 1. **b** plot of ratio of exhalation to deformed tracheal lumen area for a = 1, b = 7 muscle properties. Although there is increasing reduction in tracheal area up to 32 mm malacic segment, no instability occurs as with anterior wall property reduction. **c** plot of ratio of exhalation to deformed tracheal lumen area for a = 1, b = 1 muscle properties. Again, there is increasing reduction in tracheal area up to 32 mm malacic segment, but no instability occurs

Although there is initially a linear reduction in tracheal area under exhalation, combinations of increased malacic segment length having reduced nonlinear elastic properties often leads to an *instability* where larger, nonlinear reductions in tracheal area occur for small increases in exhalation force. Onset of instability was defined when area reduction was greater than a linear extension from the two previous exhalation force versus area ratio would predict. Onset of this instability is denoted by dotted (3 ring malacia), dash-dot (5 ring malacia) or dash (7 ring malacia) circles on graphs in Fig. 3. Physically, this means that an airway can go from patent to collapse with an increase as small as 1 Newton (0.22 lbs) or less in exhalation force. Figure 4 illustrates three steps in the 7 ring mucosa malacic where the airway goes from patent to nearly complete collapse over an increased exhalation force 0.5 to 1 Newton (0.11 to 0.22 lbs). In mechanics this highly nonlinear phenomena is known as “snap through instability”. Due to the highly nonlinear instability for the 7 ring malacic mucosa 8 and 10 mm diameter models, it was not possible to obtain a convergent solution. We believe that this instability phenomenon is the trigger point causing a transition from mild or moderate TM to severe, life threatening TM.

Increased tracheal diameter from 6 to 10 mm increased propensity for collapse (compare Fig. 2a to b to c and Fig. 2d to e to f). Increased collapse risk with increasing area is analogous to a bridge with a longer span that will deform more under the same forces unless it has more supports. If the tracheal diameter increases without a concomitant increase in tracheal mechanical properties, increased deformation is expected. Furthermore, the likelihood of instability is increased, as the instability point is pushed to even lower exhalation forces (compare Fig. 2e to f).

**Fig. 5:**
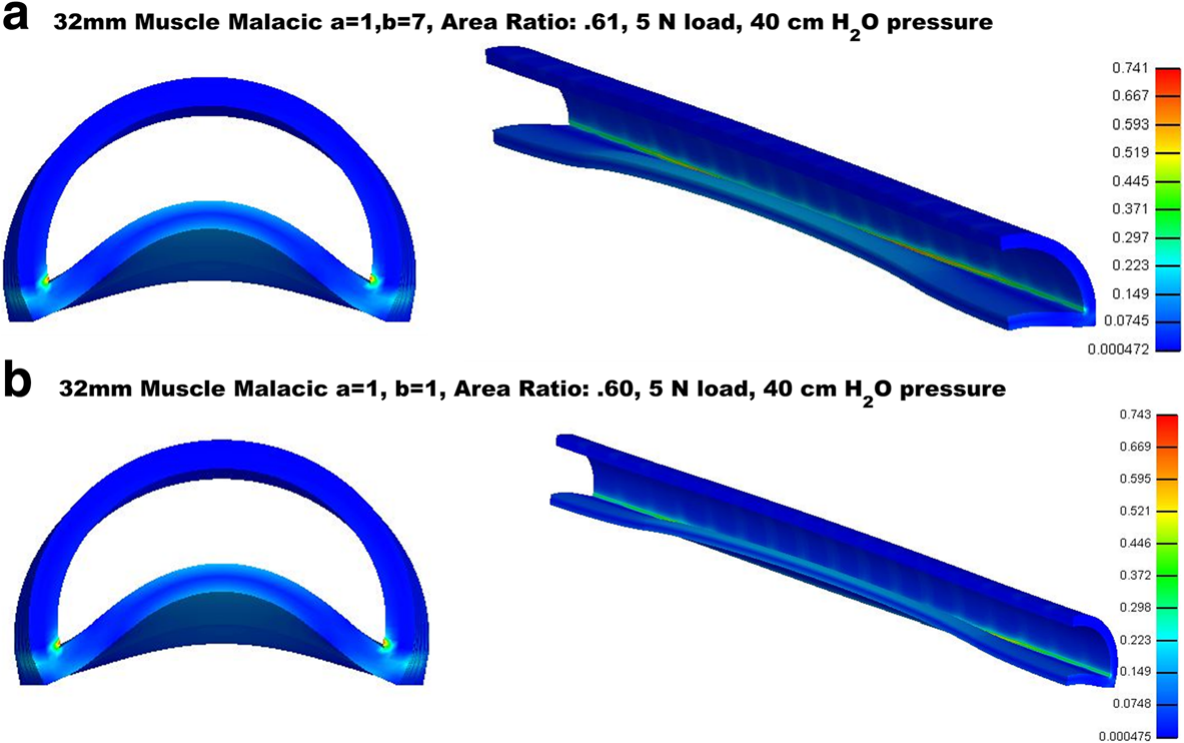
Example deformation in muscles with posterior reduced muscle malacic properties. **a** mid airway cuts showing deformation for a = 1, b = 7 properties for 32 mm softening length under maximum 5 N load. **b** mid airway cuts showing deformation for a = 1, b = 1 properties for 32 mm softening length under maximum 5 N load. Although muscle infolding occurs complete collapse is not seen as with the anterior weakening model (compare Fig. 4), airway remains patent, and tracheal wall strains are much less with a maximum of 15%

Finally, Fig. 4 illustrates reduction in tracheal area due to decreased posterior trachealis muscle nonlinear properties. Reductions in trachealis properties lead to decreased tracheal area under exhalation. However, unlike the case of reduced anterior properties, there was no instability with sudden onset of collapse. Instead, there is a continual infolding of the posterior muscle into the lumen space, but no collapse or instability (Fig. 5).

## Discussion

Computational modeling of TM supports the intriguing hypothesis that *severe TM results from nonlinear instability leading to tracheal collapse*. These same computational models suggest that risk factors for this instability leading to severe TM are large losses in anterior wall nonlinear elasticity, increased length of malacic segment, and increased tracheal diameter. The last risk factor of tracheal diameter is tightly coupled with instability, as the increased anterior wall span length increases the probability of snap through instability. Mild and moderate TM occurred in models from sequentially reduced tracheal area prior to unstable collapse.

The combination of decreased anterior wall elasticity, increased tracheal diameter and increased malacic segment length creates a “set point” of exhalation pressure at which instability is initiated and collapse occurs. Increased exhalation pressure due to activities such as coughing or higher physical exertion increases the risk of hitting the instability set point leading to collapse.

Instability is well known in the experimental and computational mechanics of elastic tubes in fluid mediums [29–31]. Indeed, post-instability buckled shapes of flexible tubes in these previous studies are similar to those in the current study. The major difference is that collapse in previous studies [29–31] occurred in circular geometry driven by increased external-internal flow pressure differential instead of focal reduction in wall properties due to malacia. However, increased pressure differential and focal wall reduced elasticity are coupled. Increased pressure differentials at some point will overcome any elastic resistance provided by the wall material, and the focal reduction in wall nonlinear elastic properties merely reduces the exhalation pressure differential necessary to initiate instability (Fig. 2).

Contrary to anterior wall malacia, posterior trachealis muscle malacia did not result in instability driven collapse, but rather exhibited a sequential area reduction without collapse. This lack of instability reflects the flat trachealis muscle geometry versus the curved anterior cartilage/fibrous tissue. The flat muscle can maintain equilibrium by sequentially deforming and stretching under exhalation. However, when the structure is curved, increasing load cannot be resisted by the material and it must rapidly snap through to a second stable equilibrium point. Thus, one would expect that snap through instability would also be a risk in bronchomalacia with an entirely circular bronchus structure.

Any computational modeling study has inherent limitations as do experimental and clinical studies. The greatest unknown is the assumed nonlinear elastic material properties and the tissue geometry. Nonlinear elastic properties were based on available experimental data for neonatal lambs [14] and engineered human mucosal tissue [26]. Such data is likely to be more relevant to TM in children than adult human tracheal tissue properties. Tissue geometry data, especially wall thickness, was chosen within literature values [23]. Finally, the degree of reduced tracheal tissue nonlinear properties due to malacia is unknown. A strength of computational modeling is that effects of postulated tissue property reductions on collapse are readily studied. However, these unknowns also point to a critical need to collect more nonlinear elastic as well as nonlinear viscoelastic data on airway tissue from a variety of species. Gathering such data on malacic airways remains extremely difficult, due to obvious difficulties in obtaining natural malacic tissue and the fact that current resection animal TM models, although useful, mimic the extreme end of TM with a complete loss of cartilage properties [9–12]. Thus, animal models that mimic the etiology of acquired TM in which mechanical pressure alters tracheal tissue morphology and nonlinear mechanical properties would be of great value.

What is the relevance of computational modeling for clinical practice? The most direct impact is that computational models reproducing salient features of TM using plausible assumptions allows rapid investigations of TM therapies, including surgical approaches, splints and stents. Such virtual testing is more cost effective for screening numerous therapies and allows treatment refinement before going into costly pre-clinical animal studies and human clinical trials during which only a limited number of therapies can be evaluated. Computational modeling also has the potential, when used in conjunction with animal models and clinical studies, to improve our understanding and diagnosis of TM.

## Conclusions

In conclusion, we applied a computational finite element modeling approach to study the effect of malacic severity (as represented by reduction in nonlinear elastic wall properties), malacic length and tracheal diameter on the risk for collapse in TM. Decreased anterior wall nonlinear mechanical properties, increased malacic length and increased tracheal diameter all increase the risks of transitioning to severe TM. The most intriguing result is that a nonlinear instability occurs at the onset of tracheal collapse, supporting the hypothesis that *nonlinear snap through instability differentiates severe from mild or moderate TM when cartilage ring nonlinear elastic properties decrease to between fibrous tissue and mucosa nonlinear elastic properties*. This hypothesis has not been noted in previous clinical reviews of TM. Of course, this hypothesis must tested in appropriate animal and clinical studies. Finally, we believe that these computational finite element models can provide further insight into evaluating surgical treatments of TM, including splints [9, 12, 32] and stents [33, 34].
